# Probiotic Potential and Safety Assessment of *Lactiplantibacillus plantarum* cqf-43 and Whole-Genome Sequence Analysis

**DOI:** 10.3390/ijms242417570

**Published:** 2023-12-17

**Authors:** Baiheng Liu, Xiaoxia Zhong, Zhiyun Liu, Xiaofeng Guan, Qi Wang, Renli Qi, Xiaorong Zhou, Jinxiu Huang

**Affiliations:** 1 Chongqing Academy of Animal Science, Chongqing 402460, China; a251211455@163.com (B.L.); zhongchuck@163.com (X.Z.); liuzhiyun2009.6@163.com (Z.L.); ggguanxf@163.com (X.G.); wangq0418@126.com (Q.W.); qirenli1982@163.com (R.Q.); zhouxiaorong429@163.com (X.Z.); 2 College of Animal Science and Technology, Southwest University, Chongqing 402460, China; 3 National Pig Technology Innovation Center, Chongqing 402460, China

**Keywords:** whole-genome sequencing, probiotics, *Lactiplantibacillus plantarum*, safety assessment

## Abstract

This study reports the whole-genome sequence of *Lactiplantibacillus plantarum* cqf-43 isolated from healthy sow feces. Based on genomic analysis, we performed a comprehensive safety assessment of strain cqf-43, using both in vitro and in vivo experiments, and explored its probiotic potential. The total genome length measures 3,169,201 bp, boasting a GC content of 44.59%. Through phylogenetic analyses, leveraging both 16S rRNA gene and whole-genome sequences, we confidently categorize strain cqf-43 as a member of *Lactiplantibacillus*. Genome annotation using Prokka unveiled a total of 3141 genes, encompassing 2990 protein-coding sequences, 71 tRNAs, 16 rRNAs, and 1 tmRNA. Functional annotations derived from COG and KEGG databases highlighted a significant abundance of genes related to metabolism, with a notable emphasis on carbohydrate utilization. The genome also revealed the presence of prophage regions and CRISPR-Cas regions while lacking virulence and toxin genes. Screening for antibiotic resistance genes via the CARD database yielded no detectable transferable resistance genes, effectively eliminating the potential for harmful gene transfer. It is worth highlighting that the virulence factors identified via the VFDB database primarily contribute to bolstering pathogen resilience in hostile environments. This characteristic is particularly advantageous for probiotics. Furthermore, the genome is devoid of menacing genes such as hemolysin, gelatinase, and biogenic amine-producing genes. Our investigation also unveiled the presence of three unannotated secondary metabolite biosynthetic gene clusters, as detected by the online tool antiSMASH, suggesting a great deal of unknown potential for this strain. Rigorous in vitro experiments confirmed tolerance of strain cqf-43 in the intestinal environment, its antimicrobial efficacy, sensitivity to antibiotics, absence of hemolysis and gelatinase activity, and its inability to produce biogenic amines. In addition, a 28-day oral toxicity test showed that the strain cqf-43 did not pose a health hazard in mice, further establishing it as a safe strain.

## 1. Introduction

Probiotics are living microorganisms known for their resilience in adverse conditions and their potential to confer health benefits upon their host [1]. The health-promoting attributes of probiotics have been substantiated through extensive research employing animal models and clinical trials. Consequently, they have garnered escalating interest in recent times. Among the diverse array of probiotics, notable examples encompass *Lactobacillus*, *Lactococcus*, *Pediococcus*, *Propionibacterium*, *Bifidobacterium*, *Bacillus*, and *Saccharomyces* [2]. When probiotics establish residence within the digestive tract, they play a pivotal role in upholding the equilibrium of intestinal microflora. They excel in preserving this balance, restraining the proliferation of harmful bacteria and contributing positively to intestinal health management and disease treatment [3]. Indeed, the incorporation of probiotics into animal diets has been recognized as a beneficial practice for enhancing the well-being of livestock and poultry. This strategy offers several advantages, including the potential to improve animal health, boost production efficiency, and curtail production expenses. Probiotics have emerged as valuable substitutes for antibiotics in the field of livestock and poultry farming [4].

*Lactiplantibacillus plantarum* is amongst the most prevalent lactobacilli species, with excellent probiotic characteristics and safety. It is widely regarded as an ideal probiotic and finds extensive application in food-ingredient processing and the formulation of microecological preparations for livestock [5]. Recent extensive research has illuminated the pivotal role of *Lactiplantibacillus plantarum* in enhancing growth performance, mitigating instances of diarrhea, and bolstering the immune resilience of livestock and poultry [6]. Paswan et al. demonstrated that dietary supplementation with *Lactiplantibacillus plantarum* (NDRI 401) positively impacted the health of rats, as evidenced by improvements in hematological parameters and blood biochemical indicators [7]. Similarly, studies by Wang et al. underscored the beneficial effects of *Lactiplantibacillus plantarum* ACCC 11016 on piglets, including enhancements in growth performance, plasma immune parameters, and the equilibrium of intestinal microbial populations [8]. Notably, these benefits were also observed when piglet diets were supplemented with heat-inactivated *Lactiplantibacillus plantarum* L-137 [9].

For a long time, *Lactiplantibacillus plantarum* has been regarded as a safe species that can be ingested by animals and humans, meeting the Qualified Presumption of Safety (QPS) criteria established by the European Union and holding a “Generally Recognized as Safe” (GRAS) status from the U.S. Food and Drug Administration (US, FDA) [10]. However, recent research has emphasized that the safety of probiotics can vary depending on the specific strain, and certain probiotics may potentially pose safety concerns, particularly for individuals with compromised immune systems [1,11]. Therefore, it is imperative to conduct more rigorous safety assessments before considering the widespread application of novel probiotic strains in large-scale food processing and the development of microbial preparations.

Advancements in sequencing technology have positioned bacterial genome sequencing as a reliable tool for assessing the efficacy and safety of probiotic strains [12,13]. Integrating whole-genome sequences with bioinformatics analysis significantly enhances our understanding of the biology of these microbes. This approach provides a comprehensive view of the genome, its diversity, and evolution of candidate probiotics, furnishing precise taxonomic and gene information relevant to probiotic traits and safety features (such as intestinal tolerance, antibiotic resistance, pathogenicity, and hemolytic activity). Noteworthy studies by Kim et al. [14] and Florez et al. [15] employed detailed genomic analyses to uncover the probiotic characteristics and safety aspects of potential probiotic strains, exemplifying the power of genomic sequencing in this domain.

To identify potential probiotics suitable for the production of livestock and poultry microecological preparations and fermented feed, we recently isolated a novel strain of *Lactiplantibacillus plantarum*, designated as *Lactiplantibacillus plantarum* cqf-43, from healthy sow feces in our laboratory. However, before considering its commercial application, it is imperative to assess the safety of this strain. In this study, we conducted whole-genome sequencing of strain cqf-43. Building upon this, we performed assessments of the strain’s safety and probiotic potential using bioinformatics analysis, in vitro experiments, and a 28-day oral feeding toxicity test. This study provides valuable information for the development of novel microecological agents for livestock.

## 2. Results and Discussion

### 2.1. Genomic Sequencing and Taxonomic Identification

**Figure 1:**
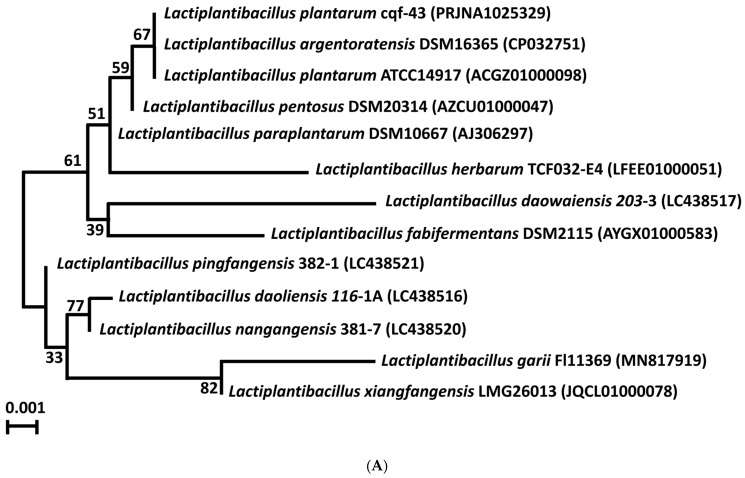

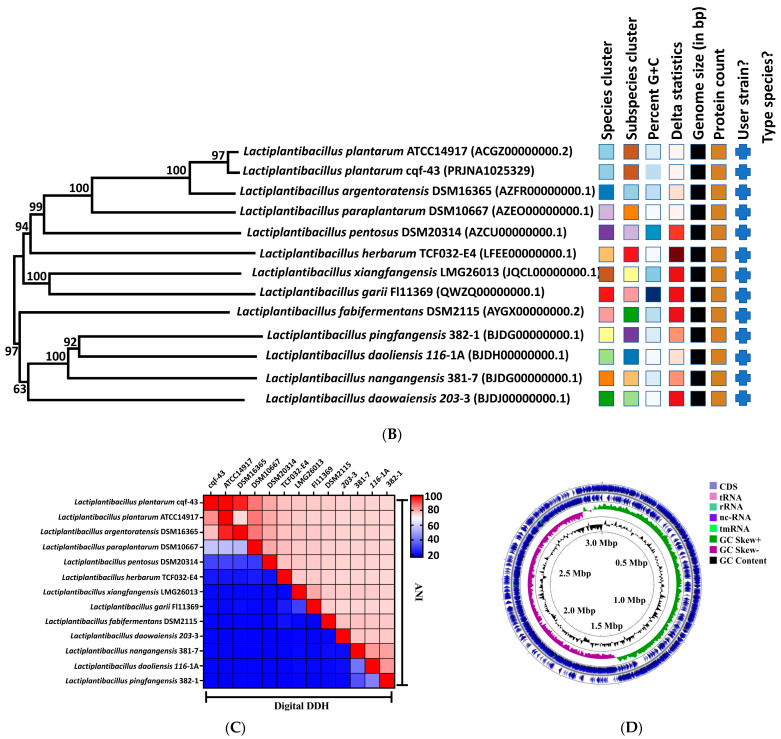
Maximum-likelihood phylogenetic trees showing the relationships between strain cqf-43 and the closely related *Lactiplantibacillus* type strains, based on the 16S rRNA gene. The numbers in the phylogenetic tree indicate the percentage of bootstrap values from 1000 repetitions (**A**). Phylogenetic comparison of strain cqf-43 with representative complete genomes of other *Lactiplantibacillus* strains carried out with the TYGS web server. The tree was inferred with FASTME 2.1.6.1. from Genome-Based Distance and Phylogeny (GBDP) calculated from 16S rDNA gene sequences. The bootstrap support value before each node represents the confidence degree of each branch. Matrix on the right (columns from left to right), dDDH species (>70%) and subspecies (>79%) cluster, strains with the same color belong to the same species or subspecies group; GC content (%), strains with the same color have similar GC content; delta values (<1) showing the tree-likeness of the data set; if a strain has an exceptionally high value, it indicates that this genome should be removed because it negatively affects phylogenetic inference; genome size in Mb; protein content; strains provided by the user (cross symbol); and type species (tick symbol) (**B**). Heatmaps showing ANI and dDDH values of strain cqf-43 and the closely related *Lactiplantibacillus* type strains (**C**). The circular genome map of strain cqf-43 is presented, comprising four distinct rings. The outermost two circles illustrate the predicted protein coding sequences (CDS), as well as the presence of tRNA, rRNA, nc-RNA, and tmRNA on both the forward and reverse strands. The third circle shows GC skews (GC skew+: green, GC skew−: purple), while the fourth circle represents GC content (in black) (**D**).

The genome of strain cqf-43 underwent high-quality complete sequencing and was taxonomically identified through both the 16S rRNA gene and whole genome-based phylogenetic analyses. In the phylogenetic analysis based on 16S rRNA gene sequences (Figure 1A), strain cqf-43 formed a distinct phylogenetic lineage closely related to both *Lactiplantibacillus argentoratensis* DSM 16365 and *Lactiplantibacillus plantarum* ATCC 14917 within the genus *Lactiplantibacillus* and showed 100% similarity in 16S rRNA gene sequences.

However, when a genome-based phylogenetic analysis was performed using TYGS, strain cqf-43 formed a distinct phylogenetic lineage with *Lactiplantibacillus plantarum* ATCC 14917, but not with *Lactiplantibacillus argentoratensis* DSM 16365 (Figure 1B). This suggests that strain cqf-43 likely belongs to the species *Lactiplantibacillus plantarum*. Furthermore, an analysis of average nucleotide identity (ANI) and digital DNA–DNA Hybridization (dDDH) values (Figure 1C) between strain cqf-43 and closely related *Lactiplantibacillus* type strains was conducted. The results revealed that strain cqf-43 shares a high ANI of 99.21% and an 83.3% dDDH value with *Lactiplantibacillus plantarum* ATCC 14917. Similarly, strain cqf-43 exhibited an ANI value of 95.58% and a digital DDH value of 80.4% compared to *Lactiplantibacillus argentoratensis* DSM16365. These values significantly surpass the established species-defining thresholds (ANI, >95%; dDDH, 70%). It is evident that strain cqf-43 shares a closer relationship with *Lactiplantibacillus plantarum* ATCC 14917, strongly indicating its classification as a *Lactiplantibacillus plantarum* strain. In contrast, *Lactiplantibacillus argentoratensis* DSM 16365 displayed high ANI and dDDH values (95.58% and 95.56%, respectively) with *Lactiplantibacillus plantarum* ATCC 14917, indicating that they share a significant genetic similarity at the species level.

### 2.2. Genomic Features

The genomic characteristics of strain cqf-43 were summarized and compared with *Lactiplantibacillus plantarum* ATCC14917 and *Lactiplantibacillus argentoratensis* DSM 16365 (Table 1). Strain cqf-43 features a single circular chromosome of 3,033,092 bp with five plasmids. In contrast, *Lactiplantibacillus plantarum* ATCC14917 has a single circular chromosome of 3,198,761 bp with 36 plasmids, and *Lactiplantibacillus argentoratensis* DSM 16365 possesses a single circular chromosome of 3,350,338 bp with five plasmids. The genomic DNA of cqf-43 has a GC content of 44.83%, encompassing 3141 total genes, 2990 CDS, 16 rRNA genes, and 71 tRNA genes. These general features align closely with those of *Lactiplantibacillus plantarum* ATCC14917 and *Lactiplantibacillus argentoratensis* DSM 16365. The GC skews and GC content play a pivotal role in understanding the genome characteristics of strain cqf-43. GC skew can be used to predict the direction of transcription and identify the coding strand in prokaryotic genomes [16]. GC content is often correlated with genome stability, and microorganisms with a high GC content may expend more energy during replication [17]. In this study, the GC skew and GC content of strain cqf-43 were in the same range as those of many *Lactiplantibacillus plantarum* and were in the same range as the remaining two strains [17,18]. The circular map of the cqf-43 genome is illustrated in Figure 1D. Further comprehensive comparative genomic analyses, particularly with *Lactiplantibacillus plantarum* ATCC14917 as a probiotic model, are important. These analyses will not only reveal the safety and distinctive probiotic effects of strain cqf-43 but also elucidate the differences between strain cqf-43 and the established probiotic standard, *Lactiplantibacillus plantarum* ATCC14917.

### 2.3. Genomic Functional Annotation

The functional annotation of the genome of strain cqf-43 was carried out using various databases, including Universal Protein Resource (UniProt) [19], Kyoto Encyclopedia of Genes and Genomes and KEGG Pathway [20], Gene Ontology (GO) [21], Protein families (Pfam) [22], Clusters of Orthologous Groups (COG) [23], the Institute for Genomic Research protein families (TIGERfams) [24], Reference Sequences (RefSeq) and Non-Redundant Protein Sequence Database (NR) (Table 2). In total, 3141 genes in the genome of strain cqf-43 were subjected to annotation. Among these, most genes were functionally annotated in the RefSeq database and NR database, comprising 2950 and 2743 genes, respectively. This accounts for 93.92% and 87.33% of the total genes, respectively. Fewer genes were functionally annotated in COG, KEGG, and KEGG Pathway, with only 1449, 908, and 868 genes, respectively.

**Figure 2:**
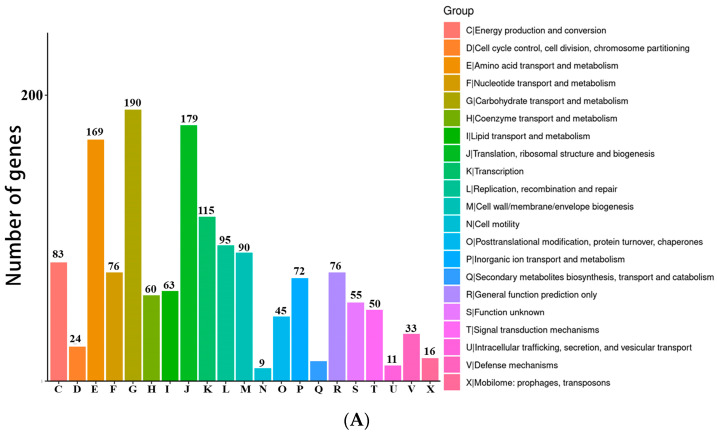

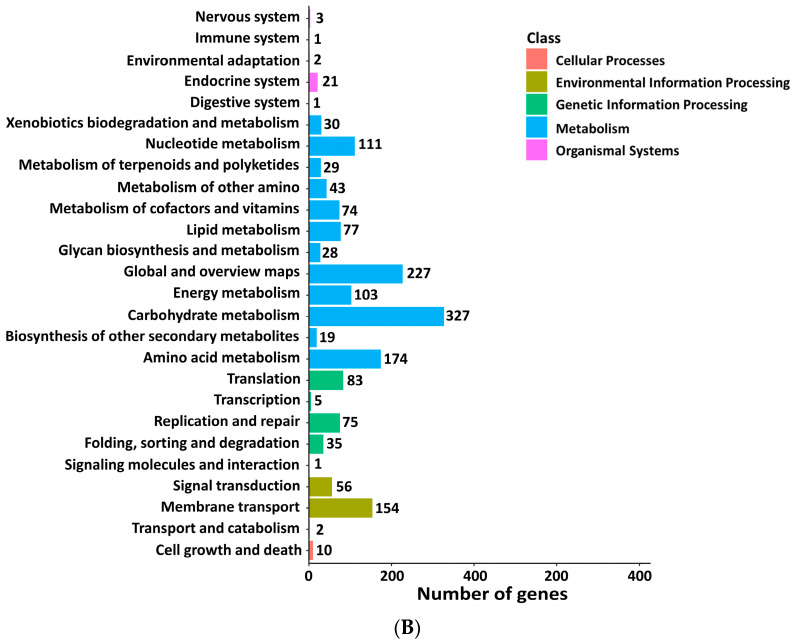
The GOC categories of strain cqf-43 (**A**). The KEGG categories of strain cqf-43 (**B**).

The functional annotation results of the COG database are shown in Figure 2A, which are divided into 21 categories. The most abundant category is carbohydrate transport and metabolism, followed by translation, ribosomal structure and biogenesis, and amino acid transport and metabolism. The functional genes annotated by KEGG database are classified into 26 KEGG functional categories (Figure 2B), which can be grouped into five major categories: cellular processes, environmental processing, genetic information processing, metabolism, and organismal systems. Metabolism is the largest category, among which the genes involved in carbohydrate metabolism are the most abundant. This trait is desirable for a probiotic strain. Similar findings were reported by Kandasamy et al. for their strain [17].

### 2.4. Mobile Genetic Element Analysis

CRISPR refers to a family of DNA repeats characterized by short and highly conserved sequences, typically found adjacent to Cas genes. When combined with Cas proteins, these repeats form the CRISPR–Cas system [14]. The genome of strain cqf-43 contains a CRISPR with associated spacer and Cas genes. Specifically, the detected CRISPR–Cas system belongs to type IIA and includes cas1, cas2, cas9, and csn2 (Table S1). The CRISPR–Cas system serves as an adaptive immune system capable of recognizing and cleaving foreign genetic material such as phages and plasmids [13,17]. Consequently, the presence of the CRISPR–Cas system in strain cqf-43 indicates its ability to effectively prevent the horizontal transfer of external virulence or antimicrobial resistance genes.

Phages serve as vital carriers for the exchange of genetic material between cells and are frequently found in the genomes of probiotic strains [17]. The prophages within strain cqf-43 were analyzed using PHASTER, revealing the presence of five phage regions (Table S2). These regions encompassed two complete phage regions (regions 1 and 2), two incomplete regions (regions 3 and 4), and one questionable region (region 5). Notably, regions 1, 2, and 5 were situated on the strain’s chromosome, whereas regions 3 and 4 were located within one of its plasmids. Specifically, regions 1 and 2 corresponded to genes from PHAGE_Lactob_Sha11 and PHAGE_Lactob_phig1e2, respectively. Region 5 matched the gene of PHAGE_Strept_315.23, while regions 3 and 4 were associated with the gene of PHAGE_Lactob_phiAT3. Since these phages are all temperate phages, it can be shown that they do not pose a threat to the safety of strain cqf-43.

### 2.5. Virulence Factors

The whole-genome analysis of strain cqf-43 was executed utilizing the Virulence Factors Database (VFDB), revealing the presence of 236 virulence factors, with identity percentages spanning from 20.31% to 75.84%. Impressively, 12 of these virulence factors exhibited identities exceeding 60% (Table S3). It is important to note that these virulence factors primarily participate in fundamental metabolic processes and are associated with the pathogen’s survival. When present in probiotics, these genes facilitate the maintenance of activity within the intestinal environment.

### 2.6. Probiotic Potential of Strain cqf-43

#### 2.6.1. Tolerance to Acid, Bile, and Heat

**Figure 3:**
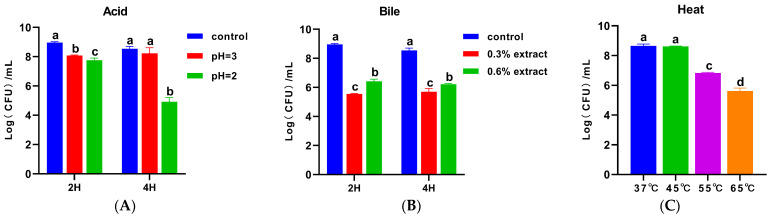
Tolerance to acid, bile, and heat of strain cqf-43. The viable cells of were counted after exposure to pH 7.5 (control), 2 or 3 for 2 and 4 h (**A**), without (control), with 0.3% or 0.6% bile salt for 2 and 4 h (**B**), and 37, 45, 55, and 65 °C for 30 min (**C**). The different letters on the error bars indicate statistically significant differences between the groups (*p* < 0.05). Each experiment was independently repeated three times.

Acid, bile, and heat tolerance are fundamental attributes of probiotics, ensuring their viability during gastrointestinal transit and processing [25]. We evaluated the survival of strain cqf-43 in simulated gastric juice and bile salt environments in vitro. As shown in Figure 3A, compared with the control group, the cell count of strain cqf-43 was significantly reduced (*p* < 0.05) after 2 h of incubation in acidic conditions (pH 2 and 3). However, following 4 h of incubation in acidic conditions (pH 2 and 3), although the cell count in the pH 2 group was significantly lower than those in the control group and the pH 3 group (*p* < 0.05), no significant difference was observed between the control group and the pH 3 group (*p* > 0.05). Given that the digestive tract averages pH 3, these results affirm the robustness of strain cqf-43 in the acidic digestive environment [26]. Under bile salt conditions, although the cell count of strain cqf-43 was significantly lower than that of the control group (*p* < 0.05), it did not completely inactivate, maintaining a substantial population of viable cells (Figure 3B). Intriguingly, it was noted that the cell count of strain cqf-43 was unaffected by the duration of culture, displaying heightened tolerance to high bile salt concentrations. These observations align with findings by Angmo et al., demonstrating that various lactobacilli possess bile salt tolerance and can thrive in bile salt-rich environments [27]. Regarding heat tolerance, Figure 3C illustrates that strain cqf-43 exhibited robust growth at both 37 °C and 45 °C, with no significant difference in cell count (*p* > 0.05). Furthermore, it retained a substantial population of viable bacteria even after exposure to temperatures exceeding 45 °C. In line with this study, Sohn et al. reported that plant lactobacilli can maintain their vitality during digestion (37 °C) and industrial processing [28].

Probiotic strains typically possess a multitude of genes encoding proteins involved in stress responses, encompassing factors like temperature, pH, and bile. These genes are intimately linked to their ability to endure the challenges of the gastrointestinal tract. In alignment with the earlier VFDB database results, strain cqf-43 was found to harbor various genes associated with gastrointestinal tolerance (e.g., *groEL*, *clpE*, *clpP*, *bsh*, and *gndA*, among others) (Table S3). These genes encode specific proteins including heat-shock proteins, molecular chaperones, and bile salt hydrolases, all of which contribute to the strain’s capacity to withstand adverse external conditions [12,14,17]. In summary, these findings indicate that strain cqf-43 likely possesses the capability to thrive under harsh environmental conditions, such as the gastrointestinal tract, while maintaining a moderate colonization ability.

#### 2.6.2. Antibacterial Activity

Once established in the intestine, probiotics can exert control over the proliferation of pathogenic microorganisms by releasing various antimicrobial substances, including organic acids and bacteriocins [29,30,31]. In our investigation, we assessed the antibacterial prowess of strain cqf-43 against common pathogenic microbes. Remarkably, strain cqf-43 showed excellent antagonistic activity against the tested bacteria, with the highest inhibition observed against *Salmonella*, followed by *Shigella dysenteriae*, and comparatively weaker inhibition against *Bacillus cereus* (Table 3). Previous studies reported the antibacterial activity of probiotics, which were consistent with this study, that *Lactiplantibacillus plantarum* had different degrees of antibacterial activity against pathogenic bacteria such as *Escherichia coli*, *Salmonella*, and *Staphylococcus aureus* [26,28]. To determine the antibacterial mechanism of strain cqf-43, the BAGEL4 webserver was used to screen for bacteriocin-related genes in the genome of strain cqf-43. The results showed that no bacteriocin-related genes were identified in the genome of strain cqf-43. However, three secondary metabolite biosynthetic gene clusters were identified in the genome of strain cqf-43 using the online tool antiSMASH. These clusters, located on the chromosome, fell into the categories of T3PKS, terpene, and cyclic lactone autoinducer families, respectively (Table S4). These families are implicated in the production of polyketides, terpenes, and cyclic lactone autoinducers, which play pivotal roles in antibacterial activities, anticancer effects, immunoregulation, and other physiological processes [12,32]. Previous studies suggested that the T3PKS, terpene and cyclic lactone autoinducer gene clusters in probiotics might be related to antibacterial activity in the intestinal environment [33].

### 2.7. Safety Test

#### 2.7.1. Antibiotic Susceptibility

The susceptibility of *Lactiplantibacillus plantarum* to antibiotics can vary significantly between strains, and when these bacteria carry transferable antibiotic resistance genes, it raises concerns about the potential transfer of virulence genes within the gut [34,35]. To gain a better understanding of the risk associated with antibiotic resistance transfer in strain cqf-43, it is necessary to study the distribution of antibiotic resistance genes in probiotics and the antibiotic resistance pattern of strains [36]. In this study, the Comprehensive Antibiotic Resistance Database (CARD) with identity >60% was used to analyze the antibiotic resistance genes of strain cqf-43. Interestingly, we identified only one antibiotic resistance gene, *poxtA*, which was located on the chromosome of strain cqf-43, posing no risk of resistance transfer (Table 4). Furthermore, the antibiotic susceptibility of strain cqf-43 was further investigated, revealing that it was sensitive to ampicillin, erythromycin, clindamycin, tetracycline, and chloramphenicol but exhibited resistance to gentamicin and kanamycin (Table 5). These findings align with previous research indicating that the majority of *Lactobacillus* strains are resistant to aminoglycoside antibiotics such as gentamicin and kanamycin, which might be attributed to the structural characteristics of *Lactiplantibacillus plantarum* cell walls [37,38,39,40]. These results underscore that strain cqf-43 lacks broad-spectrum antibiotic resistance and carries no risk of resistance transfer, aligning with the safety criteria expected of probiotics.

#### 2.7.2. Hemolytic and Gelatinase Activities and Biogenic Amine-Producing Ability

**Figure 4:**
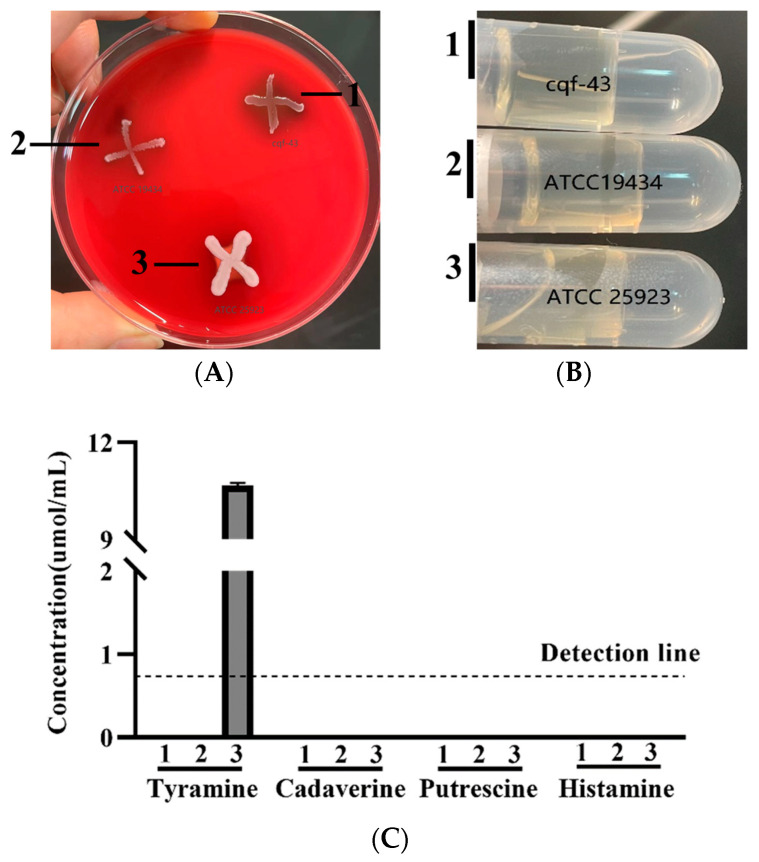
Hemolysis test (**A**); phenotypic tests (**B**); and biogenic amine production of 1. strain cqf-43, 2. *Enterococcus Faecium* ATCC 19434, and 3. *Staphylococcus aureus* ATCC 25923 (**C**). Each experiment was independently repeated three times.

Evaluating hemolytic activity, gelatinase activity, and biogenic amine-producing ability is a crucial step in screening potential probiotics for both human and animal use. While the VFDB database analysis of the genome of strain cqf-43 predicted a total of 12 virulence-related factors (with identity >60%), it is important to note that these factors primarily play roles in fundamental bacterial metabolism and are not associated with hemolysis, gelatinase production, or biogenic amine synthesis (Table S3). Probiotics should not have hemolytic activity, which is thought to lead to conditions such as anemia in the host [42,43]. The results of the hemolytic activity assay are presented in Figure 4A. A distinct hemolytic ring (β-hemolysis) was observed around *Staphylococcus aureus* ATCC 25923 colonies, while a brownish-green area was observed around strain cqf-43, indicating partial hemolysis (α-hemolysis). However, a very slight brownish-green area was also observed around *Enterococcus faecium* ATCC 19434, which exhibits γ-hemolysis, and the VFDB database indicated the absence of hemolysis-related genes in the genome of strain cqf-43. Therefore, these brownish-green areas might not be exclusively linked to the α-hemolytic activity of the test strain but could be associated with the acid-sensitive nature of the blood plate used in the experiment. Additional confirmation of the strain’s hemolytic activity through animal models is warranted. Studies have demonstrated that pathogenic bacteria can pose significant health risks to the host through their gelatinase activity and biogenic amine-producing ability [18,29,44,45]. Consistent with the VFDB database prediction results, strain cqf-43 exhibited no gelatinase activity or biogenic amine-producing ability (Figure 4B,C).

#### 2.7.3. The 28-Day Oral Feeding Toxicity Test

**Figure 5:**
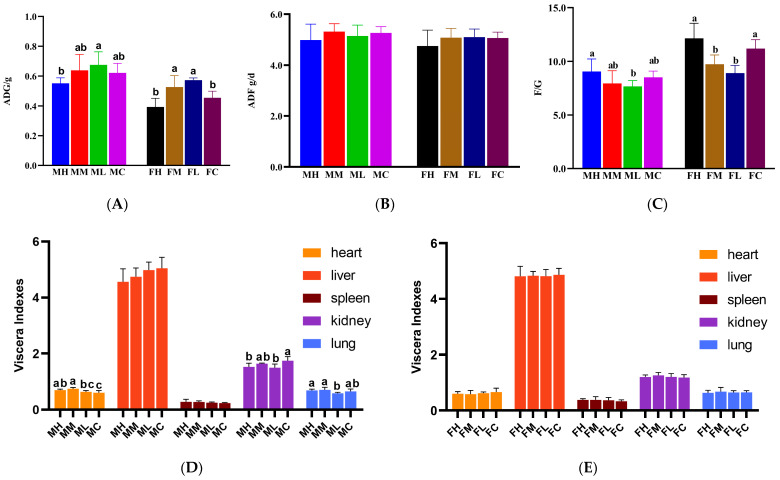
The changes in average daily gain (ADG), average daily feed intake (ADF), feed conversion (F/G), and viscera index in mice administered with strain cqf-43 via 28-day repeated toxicity (**A**–**E**). MH, MM, ML, and MC, respectively, represented high concentration (10^11^ CFU/mL), medium concentration (10^10^ CFU/mL), low concentration (10^9^ CFU/mL), and normal saline feeding groups of male mice. Additionally, FH, FM, FL, and FC had the same means in the female mice group. The different letters on the error bars indicate statistically significant differences between the groups (*p* < 0.05). Each value is expressed as the mean ± SEM (n = 6).

To further assess the safety of this strain, a 28-day oral toxicity test was conducted. Throughout the test period, mice that were continuously administered high (10^11^ CFU/mL), medium (10^10^ CFU/mL), and low (10^9^ CFU/mL) doses of strain cqf-43 via gavage remained in good health, with no general abnormal symptoms such as convulsions, diarrhea, or drowsiness observed. Key indicators, including feed intake, average daily gain, and feed conversion ratio, were monitored throughout the test period to evaluate any potential adverse effects [46]. As shown in Figure 5A–C, no significant negative effects on feed intake, body weight gain, or feed conversion ratio were observed in any of the groups during the entire testing period (*p* > 0.05). In fact, both male and female mice showed some degree of improvement in average daily gain and feed conversion ratio. Notably, the low dose (10^9^ CFU/mL) of strain cqf-43 had the most significant positive impact on growth performance. Viscera indices, which represent the relative size of the viscera and are used to assess the health status of individual animals, were calculated [47]. Figure 5D,E illustrates that the administration of strain cqf-43 did not significantly reduce viscera indices in mice in all groups, except for the lung indices of the MH and ML groups, which were significantly lower than those of the MC group. Additionally, the visceral appearance of mice in all groups did not show any pathological symptoms. These results collectively suggest that strain cqf-43 does not cause significant viscera damage. Ou et al. observed similar results when they administered *Lactiplantibacillus plantarum* WYH to mice, confirming that *Lactiplantibacillus plantarum* had no adverse effects on various viscera indices [25]. Hematological parameters were assessed (Table 6), and no significant differences in hematological parameters were observed in either male or female mice after the administration of different doses of strain cqf-43 compared to their respective control groups. This further supports the conclusion that strain cqf-43 lacks hemolytic activity. The impact of strain cqf-43 on the health of mice can be assessed by examining serum biochemical indicators [48]. Alanine aminotransferase (ALT) and aspartate aminotransferase (AST) serve as crucial markers for liver damage, while creatinine (CREA) reflects kidney function, as it is a waste product produced during muscle metabolism [49]. The serum biochemical results are presented in Table 6. Following the administration of strain cqf-43 to mice, ALT and AST levels exhibited a consistent decline compared to the control group, indicating strain cqf-43’s potential to mitigate liver damage. Moreover, male mice experienced a significant decrease in creatinine levels after gavage with strain cqf-43 (*p* < 0.05), a trend similarly observed in female mice (*p* > 0.05), suggesting a beneficial impact on kidney function. These results indicate that strain cqf-43 is generally safe in vivo and has some positive effects on animal growth.

## 3. Materials and Methods

### 3.1. Bacterial Strains, Culture Conditions, and DNA Isolation

The strain cqf-43, isolated from the feces of healthy swine and provided by the Chongqing Academy of Animal Sciences in Chongqing, China, underwent a standard isolation process. In brief, 2.5 g of feces from healthy sows were homogenized in 22.5 mL of 0.9% (*w*/*v*) sterile saline for 1 min. Serial 10-fold dilutions were performed, and 0.1-mL aliquots of the appropriate dilutions were directly spread on de Man, Rogosa, and Sharpe (MRS, Condalab, Madrid, Spain) agar plates, and incubated anaerobically at 37 °C for 48 h. Colonies with distinctive sticky filaments were selected and inoculated on MRS solid medium plates for further cultivation. Following repeated streaking and purification on MRS solid medium plates, several strains of pure culture were obtained, and one of them was designated as the cqf-43 strain.

The strain cqf-43 was cultured in MRS broth (Condalab, Madrid, Spain) at 37 °C for 16–18 h to obtain cells for DNA extraction. For DNA extraction, strain cqf-43 cells were collected by centrifugation at 8000× *g* for 4 min. Total genomic DNA was extracted from the cell pellets using SDS extraction and purified using the MicroElute^®^ DNA Clean-Up Kit (OMEGA, Norwalk, CT, USA), following the manufacturer’s instructions [12]. The purity and quantity of the isolated DNA were assessed spectrophotometrically at 260 nm using the NanoDrop^®^ One Spectrophotometer (NanoDrop Technologies, Wilmington, DE, USA).

### 3.2. Whole-Genome Sequencing of Strain cqf-43

In this study, we used the Nanopore sequencer (Oxford Nanopore Technologies, Oxford, UK) for long-read sequencing and the Illumina platform for short-read sequencing (NEBNext^®^Ultra™DNA Library Prep Kit for Illumina^®^, Ipswich, MA, USA). After sequencing, a hybrid assembly method was used to combine the short and long reads (Tsingke Biotechnology Co., Ltd., Beijing, China). An assembly pipeline for bacterial genomes, Unicycler (v0.4.9), was used to assemble and polish the hybrid sequence [50]. Assembly errors were polished using Pilon (v1.23) [51] and Racon (v1.4.16) [52]. The sequences with lengths less than 1 M were filtered and compared with the plasmid database (from NCBI) by blast. If the coverage is greater than 0.5, then it is determined as a plasmid. The complete genomic sequences of strain cqf-43 have been submitted in the NCBI database (BioProject acc. no. PRJNA1025329 and GenBank acc. nos. CP139651–CP139656). Tow genome sequences used for the comparative genome analysis were obtained from the NCBI database, including *Lactiplantibacillus argentoratensis* DSM 16365 (GenBank acc. no. AZFR00000000.1) and *Lactiplantibillus plantarum* ATCC14917 (GenBank acc. no. ACGZ00000000.2).

### 3.3. Gene Prediction and Functional Annotation

Gene prediction and computational annotation of protein-coding genes were carried out using Prokka (version 1.12) [53]. For gene function prediction, eight databases were utilized, including UniProt [19], KEGG and KEGG Pathway [20], GO [21], Pfam [22], COG [23], TIGERfams [24], RefSeq, and NR. Predicted gene sequences underwent BLAST+ (version 2.5.0+) comparisons with COG, KEGG, Swiss-Prot, RefSeq, and other functional databases to obtain gene functional annotations [54]. The software hmmer (3.3.1) [55] was used to perform functional annotation based on Pfam and TIGERFAM databases. Gene function annotation analysis such as COG and KEGG metabolic pathway enrichment analysis was performed. Additionally, the genome underwent CRISPR prediction through the Crisprcasfinder software version 1.1.2 [56] and prophage prediction using the PHASTER website [57]. Using the BAGEL4 web server and antiSMASH bacterial version 5.0, secondary metabolite biosynthesis gene clusters were identified [14,58]. To assess pathogenicity and resistance, the CARD [59] and the VFDB [60] were employed. Two search criteria were employed: a stringent search with cut-off values set at >80% identity and >60% coverage, and a less stringent search with cut-off values at >60% similarity, >60% coverage, and E-value < 1 × 10^−10^ [34]. Any identified virulence genes meeting these criteria were subjected to further investigation.

### 3.4. Phylogenetic and Genome-Related Analyses

The strain cqf-43 underwent taxonomic identification via phylogenetic analyses utilizing both 16S rRNA gene sequences and whole-genome sequences. For the 16S rRNA gene sequence-based phylogenetic analysis, alignment of the 16S rRNA gene sequences of strain cqf-43 and closely related type strains was carried out using the ribosomal database project’s fast secondary-structure-aware infernal aligner [61]. Subsequently, a maximum-likelihood (ML) tree, including bootstrap values based on 1000 replications, was constructed using the MEGA7 software Version 7.0 [62]. The similarity in 16S rRNA gene sequences between strain cqf-43 and closely related type strains was computed using the EzTaxon-e server (http://www.ezbiocloud.net/, accessed on 3 April 2022). In the genome-based phylogenetic analysis, the genomes of strain cqf-43 and closely related type strains were extracted using the TYPE GENOME SERVER (https://tygs.dsmz.de/, accessed on 10 April 2022). An ML tree was generated to establish the genome-relatedness among these strains. This assessment involved the utilization of AN and dDDH analyses. The Orthologous Average Nucleotide Identity Tool software version 0.9.0 (www.ezbiocloud.net/sw/oat, accessed on 10 September 2022) was used for ANI analysis, while DDH analysis was conducted through the Genome-to-Genome Distance Calculator version 3.0 (http://ggdc.dsmz.de/distcalc2.php, accessed on 13 September 2022) [62,63]. For these analyses, dDDH values were used as criteria for species and subspecies identification, with thresholds set at 70% and 79%, respectively, and thresholds for ANI values were set at 95–96%. These assessments were facilitated by the Type (Strain) Genome Server (TYGS) [64]. TYGS was also employed to establish a bootstrapped genome-based phylogenetic relationship among the strains.

### 3.5. Tolerance of Acid, Bile, and Heat

Stress tolerance, including resistance to acid, bile, and heat, was assessed following established methods of previous researchers with slight modifications [25]. In brief, the strain cqf-43 was cultured in MRS broth at 37 °C for 24 h, then subjected to centrifugation (8000× *g*, 10 min, 4 °C) to remove the supernatant. The bacterial pellet underwent two washes with PBS (pH = 7.4) and was finally resuspended in an equal volume of PBS (pH = 7.4). For acid and bile tolerance assessments, 0.5 mL of the bacterial suspension was added to 4.5 mL of PBS with varying pH levels (pH = 7.4, used as a control, pH = 2, pH = 3) or containing bile at concentrations of 0%, used as a control, 0.3%, and 0.6%. Incubation was carried out at 37 °C for 2 and 4 h, followed by sample dilution and enumeration. To evaluate heat tolerance, PBS (pH = 7.4, bacterial concentration of 6.5 × 10^9^ CFU/mL) containing 10% (*v*/*v*) of the bacterial suspension was exposed to different temperatures (37 °C, 45 °C, 55 °C, and 65 °C) for 30 min, followed by sample dilution and plate counting.

### 3.6. Preparation of Pathogenic Bacteria and Antibacterial Activity

The antibacterial activity of strain cqf-43 against several pathogenic bacteria, including *Streptococcus dysgalactiae* ATCC35666, *Staphylococcus aureus* ATCC25923, *Escherichia coli* ATCC25922, *Staphylococcus aureus* ATCC43300, *Listeria monocytogenes* CVCC3764, *Pseudomonas aeruginosa* ATCC27853, *Shigella dysenteriae* CGMCC1.1869, *Bacillus cereus* CVCC4101, and *Salmonella* CVCC534, was assessed following the method outlined by Saboktakin-Rizi et al., with some adjustments made as necessary [65]. An overnight culture of strain cqf-43 in MRS medium was prepared, centrifuged (8000× *g*, 10 min, 4 °C), and the supernatant was used as antagonistic substance. The pathogens were incubated in NB medium at 37 °C for 14 h and then diluted to a concentration of 10^8^ CFU/mL. These diluted bacterial suspensions were plated onto Mueller Hinton agar. Then, 100 µL of cell-free supernatant were added to the wells on the agar. After an incubation at 37 °C for 16 h, the diameter of each clear zone of growth inhibition was measured in millimeters to determine the antagonistic effects.

### 3.7. Antibiotic Susceptibility Test

Antimicrobial susceptibility was evaluated by determining Minimum Inhibitory Concentrations (MICs) of strain cqf-43 for eight antibiotics (ampicillin, vancomycin, gentamicin, kanamycin, erythromycin, clindamycin, tetracycline, and chloramphenicol). This evaluation was conducted using the broth microdilution method for antimicrobial susceptibility testing with a bacterial concentration of 10^6^ CFU/mL [34].

### 3.8. Phenotypic Tests for Hemolytic and Gelatinase Activities and Biogenic Amine-Producing Ability

To evaluate hemolytic activity, strain cqf-43 was streaked onto a blood agar plate and incubated at 37 °C for 48 h, and the hemolysis of the colony of strain cqf-43 was observed based on the zone of hemolysis around the colonies [12]. The β-hemolytic *Staphylococcus aureus* ATCC 25923 and the γ- *Enterococcus faecium* ATCC 19434 were used as control strains [66].

Gelatinase activity was determined based on the strain’s ability to degrade gelatin. Following a previously established method, strains were punctured into gelatin medium (Qingdao Haibo, Qingdao, China) and incubated at 37 °C for 4 days [67]. Subsequently, the agar plates were placed at 4 °C for 10 min, and we observed the extent of gelatin liquefaction.

The biogenic amine-producing ability of strain cqf-43 was evaluated in accordance with a method outlined by previous researchers [37]. In brief, the culture fluids of each strain were centrifuged at 8000 rpm for 10 min, and the supernatants were collected and derivatized with dansyl chloride. Then, the solutions were diluted to 5 mL with acetonitrile and completely dissolved by ultrasonic waves. After mixing well, the derived biogenic amines were quantified using high-performance liquid chromatography (HPLC, Shimadzu, Kyoto, Japan).

### 3.9. The 28-Day Oral Feeding Toxicity Test

All animal experiments were conducted in accordance with the Guide for the Care and Use of Laboratory Animals and were approved by the Ethics Committee of the Chongqing Academy of Animal Sciences (license No. 202303A). The mice, forty-eight in total (evenly split between males and females), were three weeks old on average, with an initial weight of approximately 20 g. They were acclimated to the experimental environment for 7 days. Subsequently, they were randomly assigned to one of eight groups based on their gender: 1. Male control group (MC): received gavage with physiological saline. 2. Female control group (FC): received gavage with physiological saline. 3. Male high-dose treatment group (MH): received gavage with strain cqf-43 at a concentration of 10^11^ CFU/mL. 4. Female high-dose treatment group (FH): received gavage with strain cqf-43 at a concentration of 10^11^ CFU/mL. 5. Male medium-dose treatment group (MM): received gavage with strain cqf-43 at a concentration of 10^10^ CFU/mL. 6. Female medium-dose treatment group (FM): received gavage with strain cqf-43 at a concentration of 10^10^ CFU/mL. 7. Male low-dose treatment group (ML): received gavage with strain cqf-43 at a concentration of 10^9^ CFU/mL. 8. Female low-dose treatment group (FL): received gavage with strain cqf-43 at a concentration of 10^9^ CFU/mL.

Mice were gavaged regularly every day with 0.2 mL of saline or strain cqf-43 for 28 consecutive days and weighed every other day throughout the study. Following the 28-day feeding trial, the mice underwent an overnight fasting period. On the subsequent day, they were anesthetized with CO_2_, and blood samples were collected from the eyeball for analysis of hematological parameters and serum biochemical indicators. This analysis was conducted using an automatic blood cell analyzer. The mice were humanely euthanized through spinal dislocation. The heart, liver, spleen, lung, and kidney were carefully collected, washed, and weighed to calculate organ indices.

### 3.10. Statistical Analysis

Statistical analysis was performed with GraphPad 8.0 software (San Diego, CA, USA). The data were analyzed with unpaired Student’s *t*-test or a one-way analysis of variance (ANOVA) with Dunnett’s post-test when appropriate. Mean values reported with standard error mean (mean ± SEM) were used to express all values unless otherwise indicated as appropriate. The *p* < 0.05 values denote significant differences among groups.

## 4. Conclusions

This study conducted whole-genome sequencing, in vitro assessments, and in vivo experiments to assess the safety of strain cqf-43. The results unequivocally demonstrate that strain cqf-43 is a novel strain that is safe, has strong environmental tolerance (acid, bile, and heat) and antibacterial activity, and does not have the risk of antibiotic resistance transfer, hemolytic activity, gelatinase activity, or biogenic amine production. Bioinformatics analysis and phenotypic testing of the entire genome provided support for these findings. This study introduces a novel safe strain for the future development of functional probiotics. However, to develop this strain into a widely applicable probiotic product, its probiotic properties should be comprehensively confirmed through animal experiments in subsequent studies, and its advantages should be clarified by comparing its genome and phenotype with various probiotics.

## Figures and Tables

**Table 1:** General features of the genomes of strain cqf-43.

Features	DSM 16365 (T)	ATCC14917 (T)	cqf-43
Genome size (bp)	3,350,338	3,198,761	3,169,201
GC content (%)	44.97	44.48	44.59
No. of counts	6	36	5
Number of genes	2814	3061	3141
Protein coding sequences	3191	3013	2990
tRNA	84	61	71
rRNA	16	2	16
Proteins with function prediction	2814	2779	2973

**Table 2:** General features of the genomes of strain cqf-43.

Item	Count	Percentage
All	3141	100%
Annotation	2973	94.65%
Uniprot	1788	56.92%
Pfam	2573	81.92%
RefSeq	2950	93.92%
NR	2743	87.33%
Tigrfam	1749	55.68%
GO	1712	54.50%
KEGG	908	28.91%
KEGG Pathway	868	27.63%
COG	1449	46.13%

**Table 3:** Antibacterial activity of strain cqf-43.

Type	Bacteria Name	Zone Diameter ^1^ (mm)
ATCC35666	*Streptococcus dysgalactiae*	25.67 ± 0.58 ^b^
ATCC25923	*Staphylococcus aureus*	22.67 ± 0.58 ^c^
ATCC25922	*Escherichia coli*	23.00 ± 0.50 ^c^
CVCC3764	*Listeria monocytogenes*	22.50 ± 0.50 ^c^
ATCC27853	*Pseudomonas aeruginosa*	24.67 ± 0.58 ^b^
CGMCC1.1869	*Shigella dysenteriae*	25.33 ± 0.58 ^b^
CVCC4101	*Bacillus cereus*	19.67 ± 0.58 ^d^
CVCC534	*Salmonella*	27.67 ± 0.58 ^a^

^1^ The results represent the mean ± SEM, with n = 3. ^a, b, c^ Values with different superscripts differ significantly at *p* < 0.05.

**Table 4:** Antimicrobial resistance genes identified in the genome of strain cqf-43.

Gene Name	Antibiotic Resistance Ontology Category	Target Antibiotics	Identify
*poxtA*	Antibiotic inactivation enzyme	Carbapenem, aminoglycoside, phenicol antibiotic, lincosamide, and streptogramin	100%

**Table 5:** Minimum inhibitory concentrations (MICs) of antibiotics for strain cqf-43.

Antibiotics	MIC ^1^ (µg/mL)	Cut-Off Value ^2^ (µg/mL)
Ampicillin	1	2
Vancomycin	>128	nr
Gentamicin	32	16
Kanamycin	>128	64
Erythromycin	0.25	1
Clindamycin	0.25	4
Tetracycline	8	32
Chloramphenicol	1	8

^1^ The results represent the mean ± SEM, with n = 3. NA means nonexistent circle. ^2^ Microbiological cut-off values for strain cqf-43 according to China. NR, not required [41].

**Table 6:** Hematological parameters and serum biochemical indicators in mice for 28-day repeated toxicity ^1^.

Item	MH	MM	ML	MC	FH	FM	FL	FC
Hematological parameters								
Red blood cell (RBC, 10^12^/L)	8.45 ± 0.63	8.29 ± 0.79	6.87 ± 2.51	8.13 ± 1.28	8.17 ± 0.53	7.48 ± 1.68	7.03 ± 1.08	7.57 ± 0.97
Hemoglobin concentration (HGB, g/L)	137.33 ± 8.31	136.33 ± 14.65	110.00 ± 43.49	128.33 ± 22.64	139.50 ± 8.50	126.50 ± 30.65	123.67 ± 20.7	124.67 ± 21.01
Hematocrit (HCT, %)	38.17 ± 1.64	37.18 ± 3.60	31.17 ± 11.82	35.55 ± 5.67	37.57 ± 1.77	33.82 ± 7.46	32.98 ± 5.20	34.05 ± 4.81
Mean erythrocyte hemoglobin concentration (MCHC, g/L)	359.83 ± 11.55 ^ab^	366.00 ± 6.16 ^a^	351.50 ± 15.37 ^b^	360.33 ± 9.18 ^ab^	371.17 ± 6.05	372.67 ± 9.83	373.83 ± 7.47	365.50 ± 21.82
Coefficient of variation of erythrocyte distribution width (RDW-CV, %)	15.92 ± 1.56 ^a^	14.87 ± 0.35 ^ab^	14.5 ± 0.73 ^b^	15.05 ± 0.5 ^ab^	15.17 ± 0.57	15.10 ± 0.42	15.13 ± 0.27	14.98 ± 0.56
Standard deviation of red blood cell distribution width (RDW-SD, fL)	28.73 ± 4.52	26.60 ± 1.00	26.40 ± 1.67	26.27 ± 0.73	27.90 ± 2.24	27.43 ± 1.36	28.72 ± 0.74	27.08 ± 1.35
Serum biochemical indicators								
Alanine aminotransferase (ALT, U/L)	31.80 ± 6.26	28.80 ± 5.89	41.20 ± 13.44	36.00 ± 10.79	38.00 ± 5.70	36.60 ± 10.64	38.40 ± 5.37	51.00 ± 19.65
Aspartate aminotransferase (AST, U/L)	92.40 ± 8.85	98.40 ± 16.13	88.60 ± 12.28	113.60 ± 37.53	124.00 ± 43.69	97.20 ± 18.91	98.40 ± 12.62	105.80 ± 10.45
Creatinine (CREA, umol/L)	35.80 ± 8.04 ^b^	42.2 ± 18.05 ^b^	46.20 ± 14.65 ^b^	66.60 ± 14.57^a^	44.80 ± 14.43	59.80 ± 21.82	42.80 ± 17.14	46.60 ± 26.36

^1^ Each value is expressed as the mean ± SEM (n = 6). ^a,b^ Values within a row with different superscripts differ significantly at *p* < 0.05. MH, MM, ML, and MC, respectively, represent high concentration (10^11^ CFU/mL), medium concentration (10^10^ CFU/mL), low concentration (10^9^ CFU/mL), and normal saline feeding groups of male mice. Additionally, FH, FM, FL, and FC had the same means in the female mice group.

## Data Availability

The data presented in this study are available on request from the corresponding author. The complete genomic sequences of *Lactiplantibacillus plantarum* strain cqf-43 have been submitted to the NCBI database under BioProject Accession No. PRJNA1025329, BioSample Accession No. SAMN37720970, and GenBank Accession Nos. CP139651-CP139656)).

## Supplementary Materials

The following supporting information can be downloaded at: https://www.mdpi.com/article/10.3390/ijms242417570/s1.
