# Supplementary material for: Probiotic Potential and Safety Assessment of Lactiplantibacillus plantarum cqf-43 and Whole-Genome Sequence Analysis
Source: Int J Mol Sci. 2023 Dec 17;24(24):17570. doi: 10.3390/ijms242417570 (PMC10744225; doi:10.3390/ijms242417570)
Supplement: Supplementary file 1 [file ijms-24-17570-s001.zip › ijms-2744471-supplementary.pdf]

Table S1. CRISPR-CAS system identified in the genome of strain cqf-43.

Item	Description
CRISPR ID	gnl|Prokka|assembly_1
Repeat Length	36 bp
Consensus Repeat	GTCTTGAATAGTAGTCATATCAAACAGGTTTAGAAC
Spacers Count	13
CAS-Type	cas1, cas2, cas9, csn2
Number of Crispr	1
Crispr Type	CAS-TypeIIA
Crispr Length	893 bp

Table S2. Prophage regions of strain cqf-43 identified in PHASTER

Region	Region Length (Kb)	Completeness	Total Proteins	Region Position (bp)	Most Common Phage	GC %	Classification
1	16.5	questionable	25	36009-52536	PHAGE_Strept_315.2_NC_004585(3)	42.20	Temperate
2	41.9	intact	60	1704579-1746494	PHAGE_Lactob_Sha1_NC_019489(23)	40.00	Temperate
3	71.3	intact	79	2031448-2102761	PHAGE_Lactob_phig1e_NC_004305(19)	42.05	Temperate
4	6.4	incomplete	8	12567-19027	PHAGE_Lactob_phiAT3_NC_005893(2)	40.50	Temperate
5	25.1	incomplete	14	17423-42522	PHAGE_Bacill_vB_BtS_BMBtp14_NC_048640(2)	41.95	Temperate

Table S3. Classification information for strain cqf-43 virulence gene

Subject_id	Identity	Subject_annotation	Gene function
VFG012095(gb|WP_003435012)	67.3	(groEL) chaperonin GroEL [GroEL (VF0594) - Adherence (VFC0001)] [Clostridium difficile 630]	Carbohydrate stnthesis and modification
VFG046465(gb|WP_003028672)	70.99	(tufA) elongation factor Tu [EF-Tu (VF0460) - Adherence (VFC0001)] [Francisella tularensis subsp. tularensis SCHU S4]	DNA correlated
VFG000077(gb|NP_465991)	69.79	(clpP) ATP-dependent Clp protease proteolytic subunit [ClpP (VF0074) - Stress survival (VFC0282)] [Listeria monocytogenes EGD-e]	Stress protein
VFG000080(gb|NP_464522)	60.76	(clpE) ATP-dependent protease [ClpE (VF0073) - Stress survival (VFC0282)] [Listeria monocytogenes EGD-e]	Protein synthesis and modification
VFG002162(gb|NP_465591)	67.59	(bsh) bile salt hydrolase [BSH (VF0350) - Stress survival (VFC0282)] [Listeria monocytogenes EGD-e]	bile salt hydrolase
VFG037100(gb|WP_010980745)	64.03	(msrA/B(pilB)) trifunctional thioredoxin/methionine sulfoxide reductase A/B protein [MsrAB (VF0456) - Stress survival	Protein synthsesi and modification

		(VFC0282)] [Neisseria meningitidis MC58]	
VFG002190(gb|WP_002362225)	67.51	(cpsA/uppS) undecaprenyl diphosphate synthase [Capsule (VF0361) - Immune modulation (VFC0258)] [Enterococcus faecalis V583]	Secretion system
VFG048830(gb|WP_014907233)	62.87	(gndA) NADP-dependent phosphogluconate dehydrogenase [Capsule (VF0560) - Immune modulation (VFC0258)] [Klebsiella pneumoniae subsp. pneumoniae NTUH-K2044]	De hydrogenase
VFG000964(gb|WP_010922799)	75.34	(hasC) UTP--glucose-1-phosphate uridylyltransferase HasC [Hyaluronic acid capsule (VF0244) - Immune modulation (VFC0258)] [Streptococcus pyogenes M1 GAS]	Carbohydrate synthesis and modification
VFG002182(gb|WP_002376666)	62.6	(cpsI) UDP-galactopyranose mutase [Capsule (VF0361) - Immune modulation (VFC0258)] [Enterococcus faecalis V583]	Carbohydrate synthesis and modification
VFG001967(gb|YP_002344822)	60.98	(glf) UDP-galactopyranose mutase [Capsule (VF0323) - Immune modulation (VFC0258)] [Campylobacter jejuni subsp. jejuni NCTC 11168]	Carbohydrate synthesis and modification
VFG005978(gb|WP_000686634)	75.84	(GBS_RS06585)UDP-N-acetylglucosamine--LPS N-acetylglucosamine transferase [Capsule (VF0274) - Immune modulation (VFC0258)] [Streptococcus agalactiae NEM316]	Carbohydrate synthesis and modification

Table S4. Features of gene clusters in strain cqf-43.

Region	Type	Region Position
Region1.1	T3PKS	1,673,745-1,714,914
Region1.2	terpene	2,719,620-2,740,501
Region1.3	cyclic-lactone-autoinducer	2,981,905-3,002,610
